# The Operative Treatment of Cancer with Special Reference to Cancer of the Breast

**Published:** 1894-07-21

**Authors:** 


### PROGRESS IN SURGERY.

### THE OPERATIVE TREATMENT OF CANCER, WITH SPECIAL REFERENCE TO CANCER OF THE BREAST.

If we remove the breast for cancer, the removal must be a very thorough one. No one at the present time would think of excising a cancinoma from the breast, except as a palliative proceeding expecting rapid recurrence; but too many surgeons are content simply to remove a fair amount of the breast with the growth, and to take away a very small area of skin over it, even when it is attached to the skin. Against these limited removals Watson Cheyne advances many arguments.[1] He refers to Mr. Harold Stiles' investigations, as showing how much more extensive the breast is than its prominent part; and to Sappey's researches into the course of the lymphatics, to show the necessity for free removal of the surrounding tissues. He points out that it is not enough to clear enlarged glands out of the axilla, but that the whole lymphatic area should be excised, *i.e.*, all the fatty tissue in the axilla through which the lymphatic vessels run, and the glands, very probably already infected with microscopic particles of new growth, but not yet obviously enlarged. This thorough clearance of the axilla in all cases—whether enlarged glands can be felt there or not—is a matter about which some difference of opinion exists. Treves and Butlin maintain that where we do not feel enlarged glands in the axilla, it is unnecessary to open the axilla; whilst Watson Cheyne, Volkmann, Banks, and Gross all consider that in all cases the axillary contents should be removed. Dr. Farquar Curtis, of New York, in an article on "The Cure of Cancer by Operation," says[2]: "It would seem unnecessary at this late date to dwell upon the necessity for clearing out the axilla in every case, whether the glands can be felt there or not." But many surgeons in this country do not open the axilla unless they can feel enlarged glands, and then only shell them out. How fallacious such an examination of the axilla for affected glands may be Gross shows by his list of sixteen cases, in which he could not feel enlarged glands, and yet found them on opening the axilla in fourteen. Küster also found them affected in fifty-seven cases out of sixty-five in which he could not feel them. In Gross's cases a reduction of the glandular recurrences, from 52 to 25 per cent. occurred when the axilla was cleared. There is no doubt, as Watson Cheyne points out, that after removal of the axillary fat there is some limitation of movement at the shoulder—the limb cannot be fully and freely abducted. He says, "All my patients can at least do their back hair afterwards, and I think if a patient understands that this operation gives her a much better chance of permanent recovery, very few will think that a little restriction of movement is worth considering." With regard to increased mortality from the more thorough operation, Watson Cheyne considers that if the operation is performed aseptically the mortality will be very small, and may be neglected.

What are our prospects of cure after removal of the breast for carcinoma? This question is discussed both by Watson Cheyne, and Curtis. Watson Cheyne considers that the results of operation are much better now that it is more thorough. He refers to recent French statistics, in which 25 per cent. were free from the disease after three years—cases operated on since the publication of Heidenhain's researches, by the more thorough method these researches indicated. Watson Cheyne himself began to operate in this thorough manner too late to give us as yet statistics of many cases at three years after operation, but out of fifteen cases operated on two years ago or longer only five have recurred, and of the ten in which there have been no recurrences five are three years since operation. Although in some of the cases the disease had been progressing slowly, yet in all the axillary glands were felt to be enlarged before operation, and were found diseased. But the statistics of 1,213 cases which were collected by Curtis[3]—cases operated on since 1890—give 22 per cent. of so-called cures—*i.e.*, cases free from recurrence after three years. These statistics are derived from the clinics of Czerny, Gussenbauer, Küster, Lücke, and others. The American surgeon's statistics are less carefully prepared, but indicate almost the same number of cases free from disease after three years. So that the statistics referred to by Watson Cheyne as the result of more thorough operation by the French surgeons are not much better than those of cases operated on by the old and less complete method. However, sufficient time has not elapsed for us to judge of the value of the more complete method of removal by statistics. Watson Cheyne refers to what he calls Volkmann's law, "that if, after an operation, a whole year has elapsed without recurrence, one may hope for a permanent cure; after two years this cure is probable; after three years it becomes almost certain."

With regard to the mortality after operation, Dr. Curtis gives us information. Out of his 1,213 cases already referred to there was a mortality of 6 per cent. Some American surgeons quoted by Curtis have had no deaths in a large number of cases, and Watson Cheyne only one in fifty cases.

Mr. Watson Cheyne makes some very important remarks on the mistake of watching doubtful cases of scirrhus of the breast. He thinks in all cases of doubt we should certainly make an exploratory incision, and if we are then in doubt, it will be better to remove a slice for microscopical examination. He points out that all such wounds if aseptic heal at once and give no trouble, and if the disease is cancer we do not lose favourable time.

The result of recent researches in showing the communication between the lymphatic systems of the two breasts is very interesting, as an explanation of the occasional occurrence of carcinoma in the two breasts, especially when the disease is situated on the sternal side of the breast first affected.

Mr. Marmaduke Shield[4] points out the advisability of fixing the pectoral muscle by raising the arm when we wish to ascertain if the cancerous growth is fixed to it. He thinks if the woman is thin we ought to be able to tell if the axillary glands are affected without opening the axilla, but this is not the experience of Gross already referred to.

We must now leave the subject of cancer of the breast and refer briefly to the investigations of Dr. Farquar Curtis[5] on the results of operation for cancer of other organs by operation, and first we will take cancer of the tongue. In this disease statistics are prepared from 473 cases, reported from the clinics of Whitehead, Billroth, Volkman, Czerny, König, and Kocher during the last few years. The mortality of removal of the whole tongue through the mouth without division of the jaw is 12 per cent., and by Kocher's method it is practically the same; even division or excision of a portion of the lower jaw does not materially add to the mortality. Whitehead has had a particularly high mortality in cases in which he has removed the tongue by his method and also excised glands. With regard to recurrence, one case in ten had no recurrence at the end of three years, and about one case in every three recurred during the first year, and the greater number of these in the first six months. We are not told, however, where the recurrence took place, for if it takes place in the glands, or even in the floor of the mouth, it is better for the patient than in the tongue.

There is greater difficulty in arriving at the results in operations for rectal cancer as the two methods of operating, the one from the perineum, and the more recent one of Kraske's after removal of part of the rectum, are confused. Reports of 420 cases from the clinics of Cripps and numerous German surgeons are used. One in five had no recurrence at the end of four years, and these were nearly all simply perineal operation. The mortality of both methods of operating taken together is 15 per cent.

The statistics with regard to carcinoma of the uterus seem to be of much value. The results of vaginal hysterectomy are unusually good—better than for cancer of the breast. About one in three had no recurrence at the end of three years, and 10 per cent. died from operation. Abdominal hysterectomy for cancer has a constantly decreasing death-rate, but there are not enough cases for statistics. Supravaginal amputation of the cervix gives a smaller mortality than vaginal hysterectomy, but not quite so good a result with regard to recurrence.

The mortality of excision of the larynx for cancer and the proportion of early recurrences is greater than in any other form considered in Curtis's paper.

[1] An Address on the Treatment of Cancer of the Breast, B. M. J., Feb., 1894, p. 289. [2] Medical Record, Feb. 24, 1894. [3] Ibid. [4] Clinical Journal, Jan. 10, 1894. [5] Ibid.

